# Case Report: Pediatric nasopharyngeal carcinoma masquerading as benign lymphadenopathy: diagnostic pitfalls in two cases

**DOI:** 10.3389/fped.2026.1843731

**Published:** 2026-06-16

**Authors:** Yuhan Qiu, Yan Cheng, Aiqin Lin, Hui Li, Jie Liu, Wei Zhang

**Affiliations:** 1 Department of Pathology, The First Affiliated Hospital of Wannan Medical University (Yijishan Hospital of Wannan Medical University), Wuhu, Anhui, China; 2 Department of Pediatric Surgery, The First Affiliated Hospital of Wannan Medical University (Yijishan Hospital of Wannan Medical University), Wuhu, Anhui, China; 3 Department of Clinical Medicine, Wannan Medical University, Wuhu, Anhui, China; 4 Department of Medical Biology, Wannan Medical University, Wuhu, Anhui, China; 5 Department of Education, The First Affiliated Hospital of Wannan Medical University (Yijishan Hospital of Wannan Medical University), Wuhu, Anhui, China

**Keywords:** case, EBV, Kimura disease, nasopharyngeal carcinoma, pediatric, report

## Abstract

**Background:**

Diagnosis of pediatric nasopharyngeal carcinoma (NPC) is frequently delayed due to its rarity and histologic mimicry of benign lymphoproliferative disorders.

**Case presentation:**

We report two pediatric cases of NPC presenting with isolated cervical lymphadenopathy, initially misdiagnosed as Kimura disease and classical Hodgkin lymphoma, respectively. Both patients were treated with multimodal chemoradiotherapy and remain under follow-up.

**Conclusions:**

These cases highlight the diagnostic challenges of pediatric NPC, particularly its mimicry of reactive or inflammatory conditions. Persistent cervical lymphadenopathy in children warrants early core or excisional biopsy, routine cytokeratin staining, and EBER *in-situ* hybridization to expedite diagnosis and optimize outcomes.

## Background

Nasopharyngeal carcinoma (NPC) is a malignant tumor arising from the nasopharyngeal epithelium, in which the non-keratinizing subtype is almost invariably associated with Epstein–Barr virus (EBV) infection, whereas the keratinizing form is typically EBV-negative and more commonly linked to environmental cofactors such as smoking (1). While common in adults from southern China, Southeast Asia, and North Africa, NPC is exceptionally rare in children, accounting for <1% of all pediatric malignancies even in endemic regions (2). The annual incidence in individuals under 20 years in Western countries is estimated at 0.1–0.3 per million, meaning most pathologists and clinicians will encounter at most a single case—if any—during their careers (3). This rarity, combined with the tumor's propensity for occult primary lesions and massive cervical lymphadenopathy, often results in protracted diagnostic workups focused on benign reactive, infectious, or hematologic conditions.

Childhood NPC is biologically distinct from its adult counterpart. Pediatric tumors are almost exclusively the EBV-driven non-keratinizing subtype, encompassing both differentiated and undifferentiated variants (4, 5). Both variants are characterized by dense lymphoplasmacytic infiltrates that actively remodel the neoplastic epithelium through ecological tumor-microenvironment interactions—competition and predation among cell populations gradually effacing epithelial architectural features, rendering NPC cells morphologically similar to benign lymphoid hyperplasia (6). Consequently, the histologic appearance may mimic reactive follicular hyperplasia, Kimura disease, cat-scratch disease, Langerhans cell histiocytosis, Rosai–Dorfman disease, granulomatous lymphadenitis, classical Hodgkin lymphoma (cHL), or even anaplastic large-cell lymphoma (7). It is this distinctive pathologic feature of the non-keratinizing forms that underlies the diagnostic dilemma described herein. Diagnostic confusion is compounded by the absence of systemic “B” symptoms—fever, night sweats, and weight loss—in up to 60% of children, and by the fact that laboratory markers such as elevated lactate dehydrogenase or EBV viral load are not routinely ordered by primary care physicians.

Accurate recognition therefore depends on heightened clinical suspicion and on the pathologist's readiness to perform ancillary studies—particularly broad-spectrum cytokeratins and EBV-encoded small RNA (EBER) *in-situ* hybridization—on any pediatric cervical lymph node lacking typical morphologic features of a reactive process. We describe two recent cases in which the tumor masqueraded as Kimura disease and nodular-sclerosing cHL, respectively. We detail the clinical, radiologic, and histologic findings that ultimately led to correct diagnosis, review the differential diagnoses most frequently considered in children with persistent neck masses, and provide a practical diagnostic template to shorten time-to-diagnosis and enable timely, curative therapy for this rare but highly treatable malignancy.

## Case presentation

Both patients were managed at Wannan Medical University Yijishan Hospital (Wuhu, China) between May and June 2025. Clinical data, imaging studies, and formalin-fixed paraffin-embedded biopsies were retrieved following approval by the Ethics Committees of Yijishan Hospital of Wannan Medical University (Ethics No.: 2025-280); all identifiers were removed to ensure patient anonymity. All the legal guardians of children involved in this study signed the informed consent form.

### Case 1

**Figure 1:**
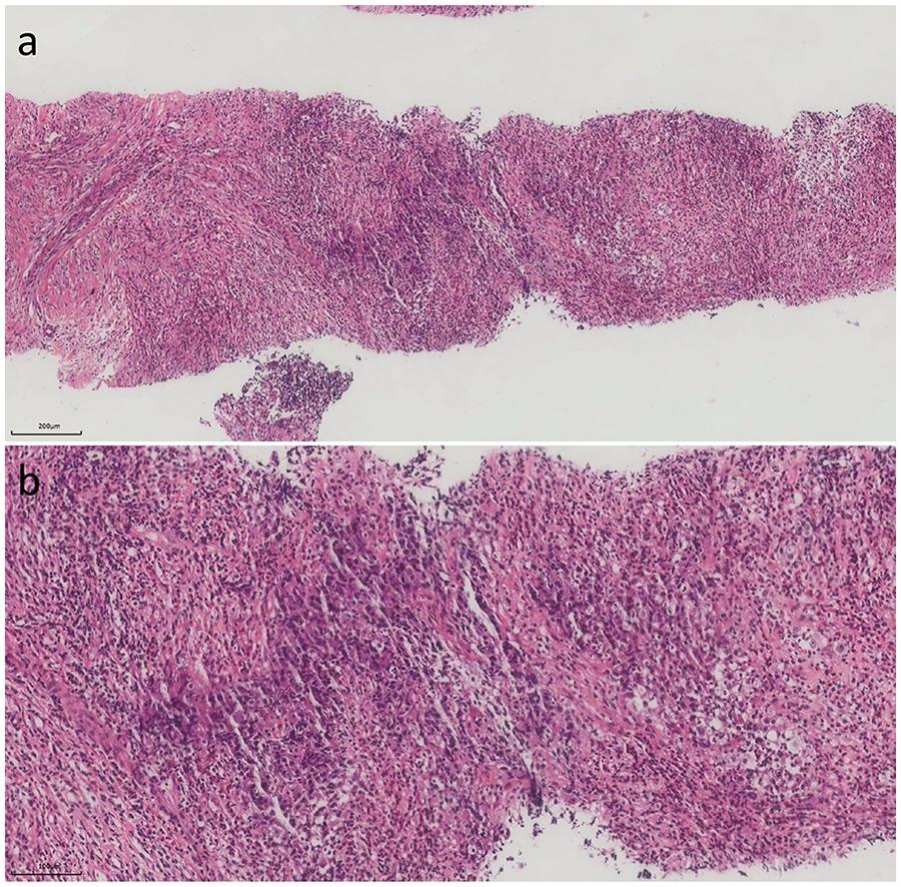
Pathological microstructure image of the first child at initial diagnosis. Core biopsy was initially reported as “eosinophil-rich granulomatous lymphadenitis, suggestive of Kimura disease” (scale bars attached). **(a)** HE staining image (10 × ); **(b)** HE staining image (20 × ).

A 12-year-old girl presented with bilateral, non-tender neck swellings. Ultrasound revealed multiple lymph nodes (largest 54 × 25 mm) with preserved hilum and hypoechoic cortex. Core biopsy was initially reported as “eosinophil-rich granulomatous lymphadenitis, suggestive of Kimura disease” (Figure 1). No response to one week of prednisolone 1 mg/kg prompted referral. Repeat 18-gauge core biopsy at our institution showed cohesive sheets of oval cells with vesicular chromatin, inconspicuous nucleoli, and moderate cytoplasm within a lymphoid background rich in eosinophils. PET/CT demonstrated hypermetabolic masses in both cervical regions and the posterior pharyngeal wall, suspicious for malignancy; possible pulmonary involvement was also noted. These findings indicated a primary nasopharyngeal lesion with cervical metastasis, rather than a benign lymphoproliferative process.

Immunohistochemistry was pivotal for diagnosis. The neoplastic cells were strongly positive for cytokeratin (CK) and positive for SALL4, while being uniformly negative for common leukocyte antigen (CD45) and a comprehensive panel of lymphoid markers (including CD20, CD3, PAX-5, CD79a). Ki-67 proliferation index was high (95%). Additional markers such as CD10, ALK, CD30, BCL-2, and MPO were negative. Extended immunostaining on deeper sections of the repeat core biopsy further showed AE1/AE3 positivity and EBER *in-situ* hybridization positivity, with negativity for p40 and p63 (Supplementary Figure S1). Combined with clinical and imaging findings, these results confirmed metastatic poorly differentiated carcinoma.

### Case 2

A 17-year-old boy presented with a right neck mass first noticed one month prior. Color Doppler ultrasound demonstrated multiple bilateral cervical lymphadenopathy, with the largest measuring 52 × 19 mm (right level II) and 31 × 16 mm (left level II). Lymph nodes showed well-defined contours, rounded morphology, intact capsules, loss of normal architecture with asymmetric cortical thickening, narrowed hilum and medulla, indistinct corticomedullary differentiation, and uniformly hypoechoic cortex. Both hilar and intranodal blood flow signals were prominent. No soft tissue swelling or abnormal echogenic lesions were observed in either neck. The thyroid gland, submandibular gland, and parotid gland were all normal in size, shape, echogenicity, and vascularity.

**Figure 2:**
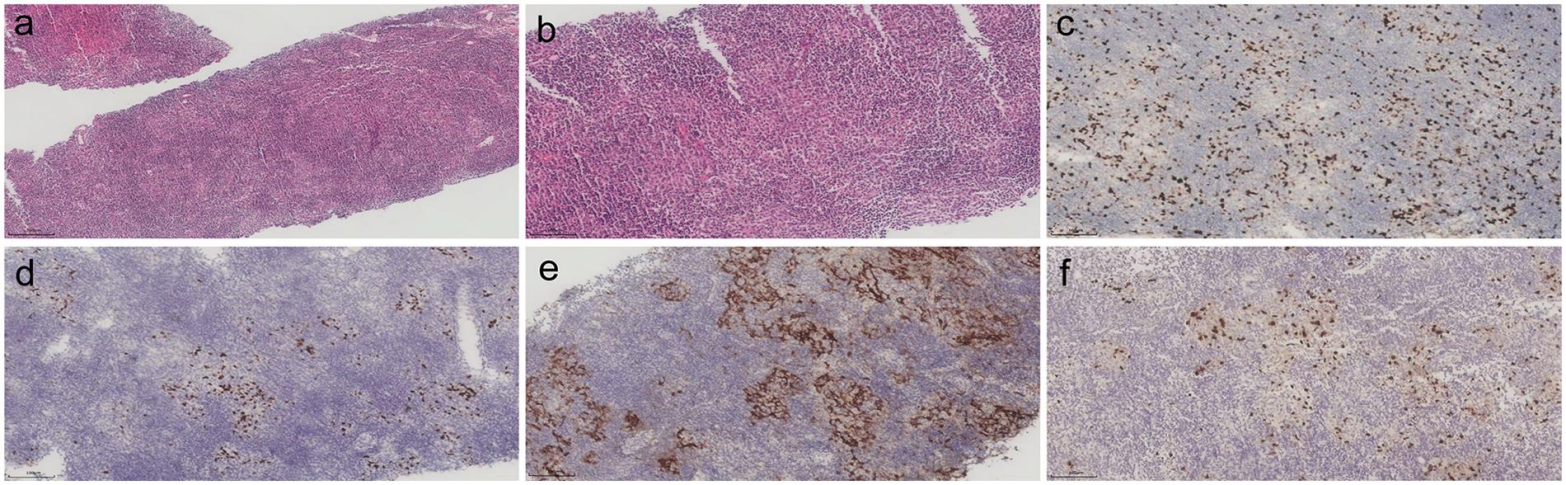
The microscopic structural image of lymph node biopsy at the initial diagnosis of the second patient (scale bars attached). **(a)** HE staining image (10 × ); **(b)** HE staining image (20 × ); **(c–f)** IHC (EnVision, 20 × ), **(c)** Ki-67 (40%+), **(d)** p40(+), **(e)** AE1/AE3(+), **(f)** EBER(+).

**Figure 3:**
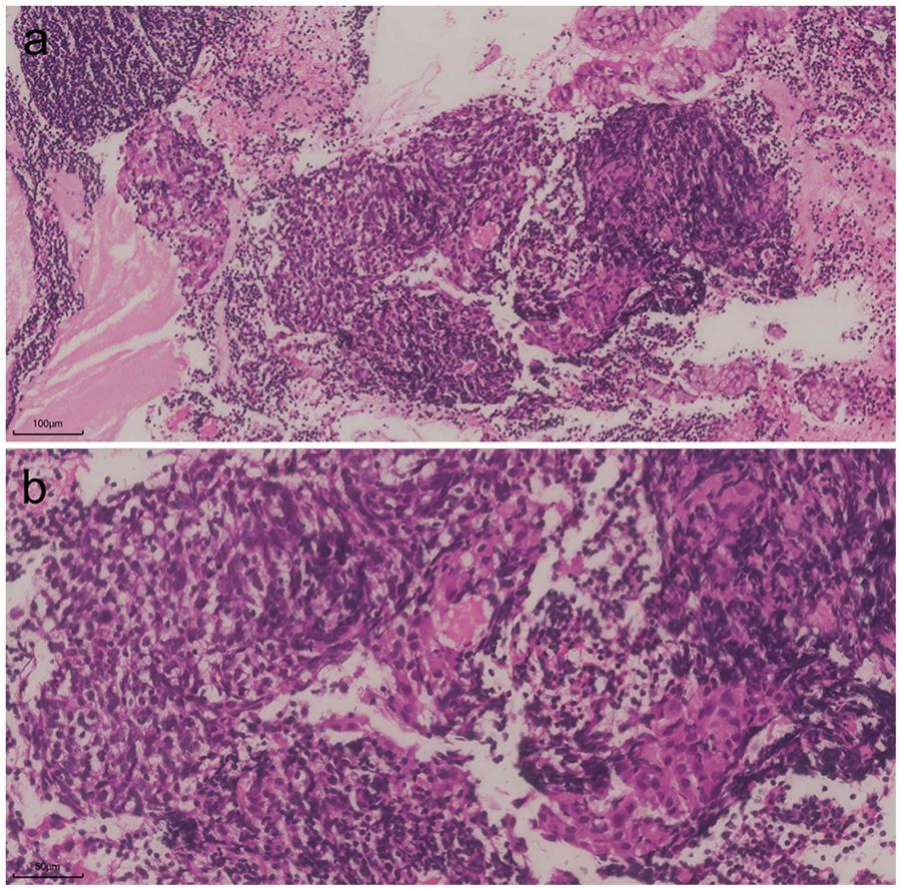
The pathological microstructure image of the nasopharyngeal mass in the second child showed small atypical cells in the lymphoid tissue (scale bars attached). **(a)** HE staining image (20 × ); **(b)** HE staining image (40 × ).

Cervical lymph node biopsy revealed cancerous tissue within the nodes. Immunohistochemistry showed: AE1/AE3(+), p40(+), Cyclin D1 (partial+), CD20(-), PAX-5(-), CD3(-), CD43(-), CD21(-), CD10(-), CD163(-), CD30(-), CD15(-), BCL-2(-), BCL-6(-), K(-), L(-), CD23(-), CD5(-), and Ki-67 (40%+). Chromogenic *in-situ* hybridization demonstrated EBER positivity (Figure 2). Based on these findings, metastatic squamous cell carcinoma was suspected, with recommendation to search for the primary lesion in the nasopharynx and other sites. Subsequent nasopharyngeal biopsy showed small atypical cells within lymphoid tissue, confirming the diagnosis of nasopharyngeal carcinoma (Figure 3).

Both patients subsequently received comprehensive chemoradiotherapy at the Children's Otorhinolaryngology Tumor Specialized Hospital. At the last follow-up (3 months post-treatment), both had completed initial therapy and showed favorable radiological response with no evidence of early recurrence.

## Discussion

Originating from epithelial tissue, NPC demonstrates significant epidemiological variation across regions, racial groups, and age categories. The teenage form of Juvenile NPC (jNPC) is particularly uncommon, representing fewer than 1% of pediatric cancers in Western nations. Although China's NPC burden is comparatively high, jNPC accounts for merely 0.1–2.3% of cases and 1%–2% of the total NPC patient pool (8, 9). Most contemporary studies define pediatric NPC as occurring in patients <18 years of age (10, 11). Both of our patients (12 and 17 years) fall within this accepted pediatric range, which is characterized by a median diagnostic age of 13–16 years across series. The tumor is vanishingly rare before age 10; incidence rises sharply during adolescence and peaks in the third decade. Boys are affected twice as often as girls, and modest but consistent familial aggregation exists—first-degree relatives of young patients have a four- to six-fold increased risk (12). Unlike adult NPC, where environmental cofactors such as salted fish consumption, cigarette smoking, and occupational formaldehyde exposure are implicated, pediatric disease appears driven almost exclusively by host genetic susceptibility and early primary EBV infection (5). Research has found that HLA-A*02:07, A*33:03, and B*38:02 alleles are associated with an increased risk of nasopharyngeal carcinoma, while HLA-A*11:01, A*31:01, B*13:01, and B*55:02 show a protective association (13). EBV DNA integration into the host genome is monoclonal in tumor tissue, arguing against a reactive process and supporting a direct oncogenic role. Circulating cell-free EBV DNA is detectable in >95% of patients at diagnosis and serves as a powerful biomarker for monitoring treatment response and early relapse (14). Despite its distinct epidemiology, the initial clinical presentation of pediatric NPC is deceptively simple, leading to the diagnostic challenges illustrated by our cases.

The classic presentation of childhood NPC is a painless, firm, upper cervical lymph node that may reach several centimeters before attracting medical attention (15, 16). In our first patient, the node exceeded 5 cm within three weeks. Retrospective series show that 70%–85% of children present with stage III–IV disease, compared with 60% of adults, indicating proportionally greater diagnostic delay in younger patients (17, 18). Furthermore, massive cervical lymphadenopathy is more common and pronounced at presentation in pediatric patients than in adults, often leading to initial suspicion of benign reactive or inflammatory conditions, as exemplified by the large nodal masses in our two cases (54 × 25 mm and 52 × 19 mm, respectively). The median symptom interval in contemporary reports is 3.5 months, but some children have waited >18 months while receiving repeated courses of antibiotics, antituberculous therapy, or corticosteroids (11, 19–21). Contributing factors include: (i) low clinical suspicion due to young age; (ii) misinterpretation of imaging—ultrasound features such as hypoechoic cortex, hilar vascularity, or even necrosis are common to both reactive and malignant nodes; (iii) empirical steroid use that may temporarily shrink nodes and reinforce impressions of inflammatory disease; and (iv) failure to obtain adequate tissue. Fine-needle aspiration yields diagnostic material in only 50% of NPC cases because the lymphoplasmacytic infiltrate masks sparse malignant islands; core or excisional biopsy is therefore mandatory when cytology is non-diagnostic.

Therefore, obtaining adequate tissue for a definitive diagnosis is paramount. Based on our experience, we propose a practical diagnostic pathway: any child with persistent cervical lymphadenopathy (>4 weeks) without an obvious infectious etiology should undergo an excisional or core needle biopsy. The biopsy specimen should be routinely subjected to immunohistochemistry for AE1/AE3 and *in-situ* hybridization for EBER, regardless of the initial histologic impression.

Kimura disease is an IgE-mediated chronic inflammatory disorder presenting as large, rubbery subcutaneous or nodal masses accompanied by peripheral eosinophilia and elevated serum IgE (22). Histology shows florid follicular hyperplasia, post-capillary venule proliferation, and interstitial eosinophilic abscesses; sclerotic late-stage lesions may contain epithelioid granulomas (23, 24). The key distinction from NPC is absence of a cohesive epithelial component. In Case 1, the initial biopsy was superficial and fragmented; eosinophil-rich granulomas were over-interpreted while small islands of cytokeratin-positive epithelium were overlooked. When clinical response to steroids failed, repeat biopsy with deeper sections and an extended immunohistochemical panel revealed the true nature of the process. Pathologists should therefore consider eosinophilic lymphadenopathy that fails to regress with corticosteroids as an indication for cytokeratin stains and EBER *in-situ* hybridization.

These cases illustrate that pediatric NPC can masquerade as virtually any lymphoproliferative or inflammatory disorder. Notably, EBER positivity is not specific to NPC, as cHL may also harbor EBV-positive lymphoid cells; therefore, the combination of cytokeratin immunohistochemistry and EBER *in-situ* hybridization is essential to distinguish metastatic carcinoma from EBV-associated lymphoma. It should also be noted that, rarely, p16-positive/high-risk HPV-associated non-keratinizing NPC may present with an AE1/AE3-positive, EBER-negative phenotype. Such cases show a trend towards more frequent involvement by HPV in recent years and should be considered when EBER is negative despite cytokeratin positivity and morphologic suspicion for carcinoma (1). In this setting, p16 immunohistochemistry and HPV DNA/RNA *in-situ* hybridization are recommended to confirm this diagnosis.

The pathologist is frequently the first to raise the possibility of carcinoma. Once diagnosed, modern multimodal therapy offers excellent cure rates, but each month of diagnostic delay translates to higher stage and potentially irreversible late effects. Successful management hinges on maintaining a high index of suspicion, obtaining adequate tissue, and interpreting results in a multidisciplinary context. Emerging artificial intelligence approaches, including deep learning-based classification of lymphoid tissue, may offer future adjunctive tools for distinguishing pediatric NPC from benign lymphoproliferative disorders (25), although prospective validation in pediatric cohorts is needed.

This report is limited by the small number of cases and its single-center origin. The absence of a primary site biopsy in Case 1 precludes direct histologic comparison between metastatic and primary lesions, though the characteristic immunophenotype (AE1/AE3+/EBER+) and favorable treatment response support the diagnosis. Longer follow-up is needed to fully assess late treatment effects and recurrence risk. Nonetheless, the diagnostic pitfalls and the proposed practical approach are widely applicable and serve as a valuable alert for clinicians and pathologists encountering similar presentations.

## Conclusion

Pediatric nasopharyngeal carcinoma is curable in >85% of cases, but its rarity and morphologic mimicry frequently lead to diagnostic delay. We recommend that any child with persistent cervical lymphadenopathy undergo core or excisional biopsy with routine cytokeratin immunohistochemistry and EBER *in-situ* hybridization. Early recognition enables timely multimodal therapy and minimizes both tumor-related mortality and treatment-associated long-term sequelae.

## Data Availability

The original contributions presented in the study are included in the article/Supplementary Material, further inquiries can be directed to the corresponding authors.

## Supplementary material

The Supplementary Material for this article can be found online at: https://www.frontiersin.org/articles/10.3389/fped.2026.1843731/full#supplementary-material

Supplementary Figure 1 Immunohistochemical staining of the repeat core biopsy in Case 1. (a) AE1/AE3 positivity (20×); (b) EBER positivity (20×).
