# Supplementary figures and images for: Case Report: Pediatric nasopharyngeal carcinoma masquerading as benign lymphadenopathy: diagnostic pitfalls in two cases
Source: Front Pediatr. 2026 Jun 16;14:1843731. doi: 10.3389/fped.2026.1843731 (PMC13314609; doi:10.3389/fped.2026.1843731)

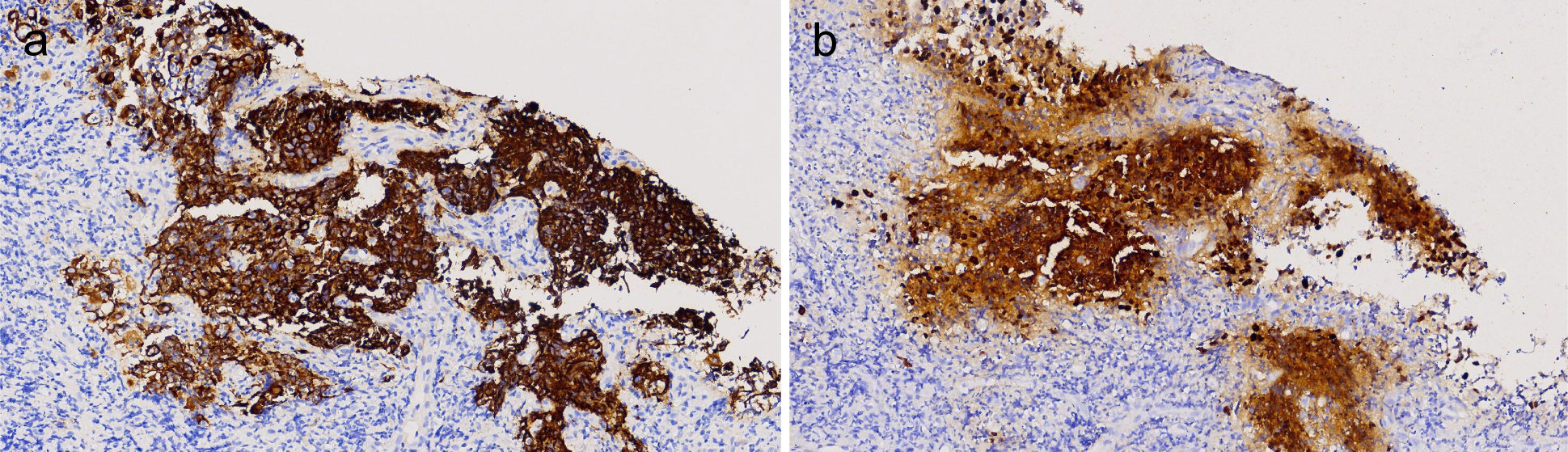

Supplement: Supplementary Figure 1 — Immunohistochemical staining of the repeat core biopsy in Case 1. (a) AE1/AE3 positivity (20×); (b) EBER positivity (20×). [file Image1.tif]
